# Ontology-based specification, identification and analysis of perioperative risks

**DOI:** 10.1186/s13326-017-0147-8

**Published:** 2017-09-06

**Authors:** Alexandr Uciteli, Juliane Neumann, Kais Tahar, Kutaiba Saleh, Stephan Stucke, Sebastian Faulbrück-Röhr, André Kaeding, Martin Specht, Tobias Schmidt, Thomas Neumuth, Andreas Besting, Dominik Stegemann, Frank Portheine, Heinrich Herre

**Affiliations:** 1 Institute for Medical Informatics, Statistics and Epidemiology (IMISE), University of Leipzig, Leipzig, Germany; 2 Innovation Center Computer Assisted Surgery (ICCAS), University of Leipzig, Leipzig, Germany; 3 Jena University Hospital, Jena, Germany; 4 GMC Systems mbH, Ilmenau, Germany; 5 SurgiTAIX AG, Herzogenrath, Germany

**Keywords:** Perioperative risks, Ontology, Risk definition, Risk specification, Risk identification, Risk analysis, Agent system

## Abstract

**Background:**

Medical personnel in hospitals often works under great physical and mental strain. In medical decision-making, errors can never be completely ruled out. Several studies have shown that between 50 and 60% of adverse events could have been avoided through better organization, more attention or more effective security procedures. Critical situations especially arise during interdisciplinary collaboration and the use of complex medical technology, for example during surgical interventions and in perioperative settings (the period of time before, during and after surgical intervention).

**Methods:**

In this paper, we present an ontology and an ontology-based software system, which can identify risks across medical processes and supports the avoidance of errors in particular in the perioperative setting. We developed a practicable definition of the risk notion, which is easily understandable by the medical staff and is usable for the software tools. Based on this definition, we developed a Risk Identification Ontology (RIO) and used it for the specification and the identification of perioperative risks.

**Results:**

An agent system was developed, which gathers risk-relevant data during the whole perioperative treatment process from various sources and provides it for risk identification and analysis in a centralized fashion. The results of such an analysis are provided to the medical personnel in form of context-sensitive hints and alerts. For the identification of the ontologically specified risks, we developed an ontology-based software module, called Ontology-based Risk Detector (OntoRiDe).

**Conclusions:**

About 20 risks relating to cochlear implantation (CI) have already been implemented. Comprehensive testing has indicated the correctness of the data acquisition, risk identification and analysis components, as well as the web-based visualization of results.

## Background

Patient safety is a quality objective and an important factor of the quality of treatment in hospitals in general [1]. Prevention of medical errors and risks is a significant method to improve patient safety. Medical personnel often work under great physical and mental strain. In medical decision-making, errors can never be completely ruled out [2]. In 2000, the report “To Err is Human” [3] was published by the Institute of Medicine of the US National Academy of Sciences (IOM). This attracted great international attention and moved the topics of medical risks, errors and patient safety into the focus of the scientific interest. The IOM concluded in the report that from 2.9 to 3.7% of all patients admitted to hospitals in the USA sustain an adverse event. In 70% of these cases, the patient retains no or only minor damage, 7% lead to permanent damage and 14% cause the patient’s death. The study also showed that between 50 and 60% of these adverse events could have been avoided through better organization, more attention or more effective security procedures. Analyses show that the number of medical errors in Germany is also not negligible. According to a report by the Robert Koch Institute [4], the incidence of suspected medical errors is approximately 40,000 cases across the country per year. Hence, the estimated error recognition rate of 30% corresponds to the rate of approximately 12,000 recognized medical errors per year.

Since the publication of “To Err Is Human”, risk management and patient safety has consistently remained a topic of interest for scientific studies as well as for suggestions of goals for improvements [5]. Critical situations arise especially during interdisciplinary collaboration and the use of complex medical technology, for example during surgical interventions and in perioperative settings. Especially the oversight of medically relevant treatment data or an incomplete medical history may lead to incorrect treatment [6].

We present an ontology and a conception for an ontology-based software tool, which can identify and analyze risks across medical processes. Furthermore, the tool supports the avoidance of errors in the perioperative setting. The results of the risk analysis are conveyed to medical personnel in form of context sensitive hints and alerts. The software architecture is designed to respond not only to risks within a single treatment step, but to also consider the patient’s entire stay in the hospital. For a practical implementation in the clinical environment, the cochlear implantation (CI) was selected as a surgical use case at Jena University Hospital. For this purpose, medical and technical treatment risks were analyzed and medical guidelines and standards were taken into account. In addition, data and information sources were defined based on an anonymized CI patient record. Further sources of critical events were collected by undertaking of qualitative interviews with technical, nursing and medical personnel participating in a CI treatment process. On this basis, risk situations were defined and integrated into ontological models. This work is a part of the OntoMedRisk project [7] funded by the German Federal Ministry of Education and Research.

## Methods

### Introduction in General Formal Ontology (GFO)

The development of the intended ontologies and of the needed ontological analyses are carried out within the top-level ontology GFO [8, 9]. In GFO, the entities of the world are classified into categories and individuals. Categories can be instantiated, but individuals are not instantiable. GFO allows for categories of higher order, i.e. there are categories whose instances are themselves categories, for example the category “species”. Spatio-temporal individuals are classified along two axes, the first one explicates the individual’s relation to time and space, and the second one describes the individual’s degree of existential independence.

Spatio-temporal individuals are classified into continuants, presentials and processes. Continuants persist through time and have a lifetime. A particular kind of continuant corresponds to ordinary objects such as cars, balls, trees, etc. These are called material objects: they carry a unity, consist of matter and occupy space. The lifetime of a continuant is presented by a time interval of non-zero duration; such time intervals are called chronoids in GFO [10]. Continuants are individuals, which may change, for example, an individual cat *C* crossing the street. Then, at every point in time *t* of crossing, *C* exhibits a snapshot *C(t)*. These snapshots differ in their properties. Further, the cat *C* may lose parts while crossing, though, remaining the same entity. The entities *C(t)* are individuals of their own, called presentials; they are wholly present at a particular point in time, being a time boundary. If the continuant is a material object *M*, the presentials exhibited by *M* at point in time *t*, denoted by *M(t)*, are called material structures. Presentials cannot change, because any change needs an extended time interval or two coinciding time boundaries.

Processes are temporally extended entities that happen in time, for example a run; they can never be wholly present at a point in time. Processes have temporal parts, being themselves processes. If a process *P* is temporally restricted to a point in time then it yields a presential *M*, which is called a process boundary of *P* [10]. Hence, presentials have two different origins, they may be snapshots of continuants or parts of process boundaries [9]. There is a duality between processes and presentials, the latter are wholly present at a point in time, whereas this is never true for processes. The corresponding classes/sets of individuals, denoted by the predicates *Cont(x)*, *Pres(x)*, and *Proc(x)*, are assumed to be pair-wise disjoint. Processes are the most basic kind of entity, because they form a ground for presentials and continuants, and determine the coherence of spatiotemporal reality. A boundary of a process *P* is defined by the restriction of this process to a point in time of its temporal extension. We postulate that any presential is a part of some process boundary.

The integration between material objects and processes is proposed in the integration law in GFO, which states that for every material object *M*, being a continuant, there is a process *Proc(M)*, the boundaries of which coincide with the presentials exhibited by *M*. There are several basic relations which canonically connect processes, presentials, and continuants [8, 9].

Spatio-temporal individuals, according to the second axis, are classified with respect to their complexity and their degree of existential independency. Attributives depend on bearers, which can be continuants, presentials, and processes. Situations are parts of reality, which can be comprehended as a coherent whole [11]. Material situations are composed of material objects, which are connected by relators, and relators are instances of relations. Situoids are processes, which satisfy principles of coherence, comprehensibility, and continuity. A surgical intervention is an example of a process or a situoid. A snapshot of this situoid at a certain point in time is a surgical presentic situation, which has a spatial location and includes various entities such that a coherent whole is established.

There is a variety of types of attributives, among them, qualities, roles, functions, dispositions, and structural features. Properties are categories, the instances of which are attributives. According to the different types of attributives (relational roles, qualities, structural features, individual functions, dispositions, factual, etc.) we distinguish quality properties and role properties, and the role properties are classified into relational role properties (abr. relational properties) as well as social role properties (social properties).

### Ontological definition of the risk notion

The solution of all philosophical problems related to the notion of risk is out of the scope of this paper. Instead, we focus on a practicable definition of the risk notion, which can be easily understood by medical staff and is usable for the software tools. Our definition of the risk notion has been developed in close cooperation with domain experts (medical staff). Based on this definition, it should be possible for the medical staff to specify the relevant risk types, and for the software to identify and to analyze the risk in a particular treatment situation.

There are various definitions of the notion of risk. One of the most known/popular definitions is presented in [12]. The authors divide the notion of risk into three components, which are associated to the following questions:

1. What can happen, i.e., what can go wrong? (scenario)
2. How likely is it that that will happen? (probability of the scenario)
3. If it does happen, what are the consequences? (consequence of the scenario)

A risk, then, is a triple which consists of a scenario, the probability of that scenario, and consequence of that scenario.

Furthermore, there are several standards investigating the notion of risk. The ISO/IEC 27005:2008 [13] defines the notion of risk (information security risk) as “*potential that a given treat will exploit vulnerabilities of an asset or group of assets and thereby cause harm to the organization*”; the OHSAS 18001:2007 [14] - as a “*combination of the likelihood of an occurrence of a hazardous event or exposure(s) and the severity of injury or ill health that can be caused by the event or exposure(s)*”; and the ISO 31000 (Risk management) [15] - as an “*effect of uncertainty on objectives*”.

In [16] the authors analyze 11 common definitions of risk and characterize them based on three categories: (a) risk as a concept based on events, consequences and uncertainties; (b) risk as a modeled, quantitative concept (reflecting the aleatory uncertainties); and (c) subjective risk descriptions. Most definitions belong to category (a), the rest can be interpreted both in the sense of (b) or (c).

The common ground of most risk definitions is that all of them consider a risk as involving a possibility for the occurrence of a particular event or situation. Most of these definitions consider such events as adverse ones.

The ontological analysis of risk is carried out within the framework of GFO and takes into account the available definitions of risk. The analysis is built upon the ontology of situations and situation types, which partly uses ideas presented in [11, 17]. Adverse situations are situations that contain adverse events. In this paper we use the notion of adverse event/situation not only in the sense of “*Any untoward occurrence that may present during treatment with a pharmaceutical product but which does not necessarily have a causal relation to the treatment*” [18], but we also include events/situations that are not related to medical interventions.

The notion of a possible situation is established within the framework of a particular actualist representationism, which postulates that possible situations are abstract entities, the existence of which is consistent with the currently available knowledge about the actual world. This view is partly influenced by [19–21] and is subsequently explicated for material situations. Material situations are composed of material facts, which are constituted by material objects and connecting relators. An example of a material fact is a spatio-temporal entity that is denoted by the expression “John’s drinking a beer”. Associated to this fact we may construct the relational proposition “John is drinking a beer”. There is a difference between a fact and the corresponding proposition. A proposition is an abstract entity, which can be satisfied by facts (which are parts of reality). Arbitrary abstract situations are sets of relational propositions, which are not necessarily abstracted from real, i.e. actual situations. An abstract situation S is realized by an actual situation S′ if any relational proposition in S is satisfied in the situation S′. An abstract situation S, related to a domain D, is said to be possible if it is consistent with the currently available knowledge about D, the domain experts agreed on. Hence, a possible situation has the potential to be realized by an actual situation. A (spatiotemporal) situation S is said to be a risk situation if it satisfies certain conditions, which imply that for one of its possible succeeding situations S′ any of its realizing situations is an adverse situation.

We hold that a risk exists in a situation, that it depends on it, and, hence, that it can be considered as a situation’s property. We distinguish between single (in sense of gfo:Property [8]) and composite properties, the latter being composed of single ones and which can be disassembled by the relation gfo:has_part.

**Definition 1.** A composite property *CP* is a property that has as parts several single properties *SP1*, ..., *SPn*.

**Definition 2.** A risk for an adverse situation of type *AST* is a composite property *CP* such that every situation *S* possessing the property *CP* has a possible succeeding situation of type *AST,* which can be realized with a certain probability.

**Definition 3.** A risk is a composite property *CP* for which there exists an adverse situation *AST* such that *CP* is a risk for the adverse situation *AST* (as defined by 2).

**Fig. 1:**
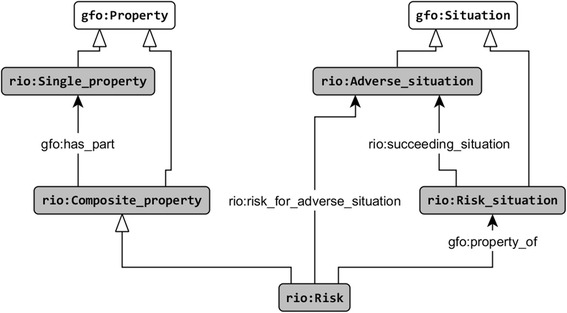
Definition of the risk notion (the white arrows represent the is-a relation)

**Definition 4.** A risk situation is a situation having at least one risk (Fig. 1). In this paper, we consider risk situations as situations with a risk recognized as relevant by the medical community and non-risk situations as situations with no risk recognized as relevant by the medical community.

**Example 1.** The risk of a bacterial infection during cochlear implantation in infants depends on various parameters, such as the infants’ age, the corresponding bone thickness of the skull and the inner ear structure. If the child is younger than 5 months, the bone thickness mostly remains below 2 mm. Thus, the risk of penetrating the skull and injuring the dura mater during surgery increases so that the risk of the bacterial dura mater infection (meningitis) increases as well. The ground-truth probability for the adverse event of dura mater infection during CI is about 5–9% [22]. For meningitis prevention, the patient has to be vaccinated against pneumococcus, meningococcus and haemophilus influenzae type b several weeks before the surgery (indication phase). In addition, an antibiotic prevention should be performed right before the surgery. According to our definition an increased risk for acquiring meningitis can be represented as a composite property, consisting of three single properties, namely, the young age (< 5 month), the absence of a meningitis vaccination, as well as the absence of an antibiotic prevention. This example is used in this paper for further explanations.

## Results

### Risk Identification Ontology (RIO)

**Fig. 2:**
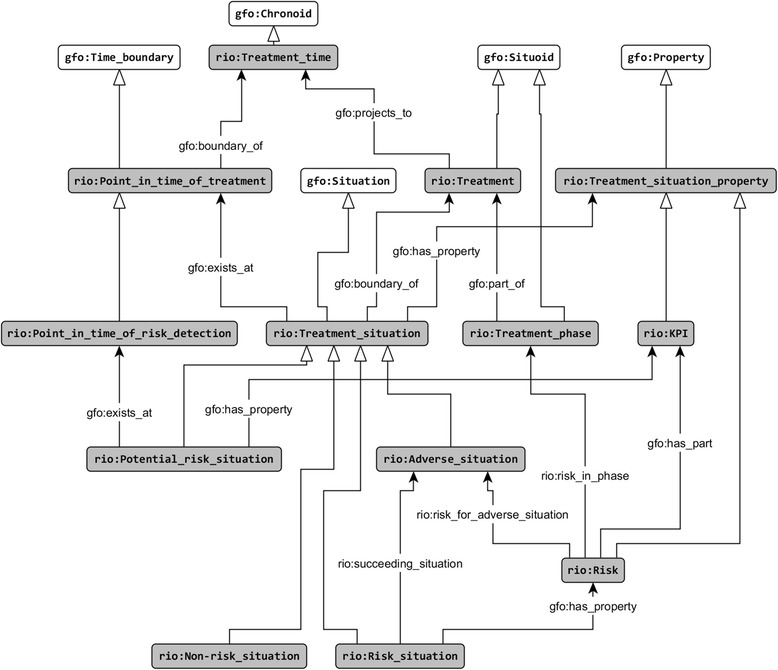
Risk Identification Ontology (RIO)

We developed a Risk Identification Ontology (RIO, Fig. 2), which is built upon the ontological model of the notion of risk. This ontology is used for the specification and the identification of perioperative risks. The ontology RIO is founded in the GFO. As the starting point we consider the treatment process, which may consist of various treatment phases (gfo:has_part). The complete treatment as well as the phases are complex processes (gfo:Situoid). The treatment has a particular temporal extension, called the treatment time (gfo:Chronoid). According to GFO processes are projected (gfo:projects_to) onto their time intervals. For every point in time (gfo:Time_boundary) of the treatment exists (gfo:exists_at) exactly one treatment situation (gfo:Situation). A point in time of the treatment is according to GFO a boundary of the treatment time (gfo:boundary_of), whereas the corresponding treatment situation is a boundary of the treatment itself.

For each treatment phase, particular points in time of risk detection (PTRD) can be defined. The treatment situations, existing at these points in time, are analyzed with respect to the existence of risks. Such situations are called potential risk situations (PRS), because they do not necessarily contain risks. Situations and in particular treatment situations possess various properties (gfo:Property). These properties may belong to the situation, but also to the participants, as, for example physicians (doctors), medical instruments, and, most important, to the patients. We consider these properties also as properties of the current treatment situation (gfo:has_property). Properties of the potential risk situations that are relevant for the estimation of the risk are called KPIs (Key Performance Indicators) in this paper. According to Definitions 1–4 a particular combination of a subset of the KPIs of a PRS (for example, age of patient = 3 months, menginitis vaccination = false) is a risk if the PRS may lead to an adverse situation at a later point in time (rio:succeeding_situation).

A PRS may contain various risks, and risks of the same type (the instances of the same risk class) may occur in distinct PRS and may lead (rio:risk_for_adverse_situation) to distinct adverse situations (the instances of the same adverse situation class). Each KPI is associated with potential risk situations, whereas the risk situations additionally possess the composite risk properties. Furthermore, the risks can be related to those treatment phases for which they are relevant (rio:risk_in_phase). A risk is relevant in a particular phase, if all required KPI values for the risk assessment need to be recorded (e.g. according to external or internal hospital guidelines) and need to be available in this phase in a respective database to prevent the risk from being realized in an adverse situation. Adverse situations may exhibit various degrees of severity and risks may possess various probabilities for the occurrence of adverse situations.

With help of the RIO the risks in a current potential risk situation are identified by the software component OntoRiDe, and, hence the situation can be classified either as a risk or as a non-risk situation.

### Risk specification

#### Perioperative risk assessment

For the development of a perioperative risk identification ontology the recognition and assessment of potential medical, technical, organizational, and human risk factors are an essential prerequisite. Therefore, an extensive risk assessment was performed for an otorhinolaryngological use case. The insertion of cochlear implants (CI) was chosen in order to demonstrate the features and benefits of the ontology-based risk identification system. The perioperative medical and technical risk factors, procedure related complications and their complication rates as well as prevention strategies were extracted from peer-reviewed publications and evidence-based best-practice guidelines of the German Society of Oto-Rhino-Laryngology, Head and Neck Surgery [23]. In addition, entries of the Critical Incident Reporting System (CIRS) of the University Hospital Jena (Germany) and an example of an anonymized patient record were analyzed for organization and human-related risk assessment. The derived risk characteristics, potential following adverse situations and their causes were used to describe relevant perioperative and cross-process risks factors.

#### Perioperative process modeling

**Fig. 3:**
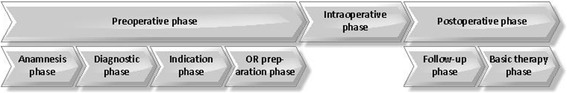
Treatment phases

The information of risk factors and of potentially adverse events has to be provided to the responsible medical personnel at the right time by offering appropriate context-sensitive hints and alerts. Therefore, the medical and organizational processes have to be taken into account. The general perioperative workflow of the CI treatment was modeled and visualized in a process diagram, as event-driven process chain (EPC). In the following, both generalized and use-case specific treatment phases were defined in the formal process model. The generalized treatment phases are depicted in Fig. 3. Besides the CI treatment process, the defined phases are suitable for representing various elective surgeries and interventions.

The treatment process was modeled by representing the sequence of clinical activities, treatment decisions, parallel processes and possible events, the involved persons as well as resources, like data and documents, medical devices, or IT systems. In addition, the identified risk factors, complications, and prevention activities were integrated in the process model.

By mapping the identified risk factors to the dedicated activities and treatment phases, the process model was then used subsequently for further risk assessment and perioperative risk modeling. This enabled over 120 potential perioperative risks to be identified and also mapped to their related process step in the process model.

#### Perioperative risks modeling

In the next step the identified potential risk factors, adverse situations, and critical incidents, which are related to cochlear implantation interventions, were examined in an extensive risk analysis. Thereof, a risk classification for formal risk specification was derived. The identified risk factors were subsequently classified into different categories of medical, organizational, technical, or human-related risks. Thus, the treatment phases were categorized into risk detection phases, in which the corresponding risk is relevant and could potentially lead to an adverse situation. Additionally, there is a category for cross-process risks, which could lead anytime to an adverse situation, e.g. the risk of dizziness and falls or the high bleeding risk during surgery due to anticoagulant medication.

**Fig. 4:**
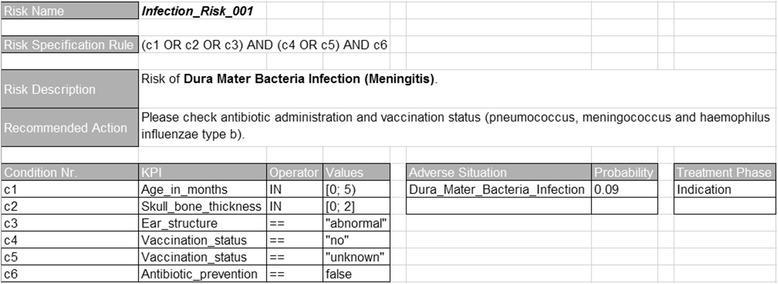
Risk specification

For each treatment phase, different KPIs were defined, which allow the identification of specific perioperative risks. The KPIs are linked with operators and a certain data range to a conditional expression of a possible risk factor (e.g., c1: Age_in_months IN [0, 5), c4: Vaccination_status == “no”, Fig. 4, Example 1). The KPI data type values could be for instance a Boolean value, text, date, or number. A combination of these conditional expressions is formalized as a risk specification rule. If the risk specification rule becomes true, due to the values of their conditions and KPIs, there is a high occurrence probability of adverse situations, which have to be also specified for each risk. In addition, for each adverse situation an occurrence probability and a severity were defined (the severity is defined on a separate spreadsheet). In the risk specification, the KPIs were described along with their possible acquisition sources. Therefore, the risk specification defines both the required measurement phases and the measurement sources, like patient-related data and sensor data, e.g. data from the digital patient record, the hospital information system, checklists, or situations in actual process execution. In Fig. 4 a risk specification based on Example 1 is presented.

**Fig. 5:**
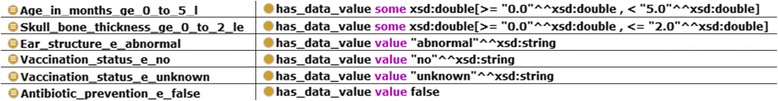
Risk conditions

**Fig. 6:**
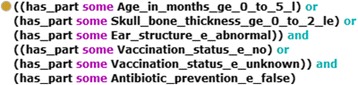
Risk specification rule

**Fig. 7:**
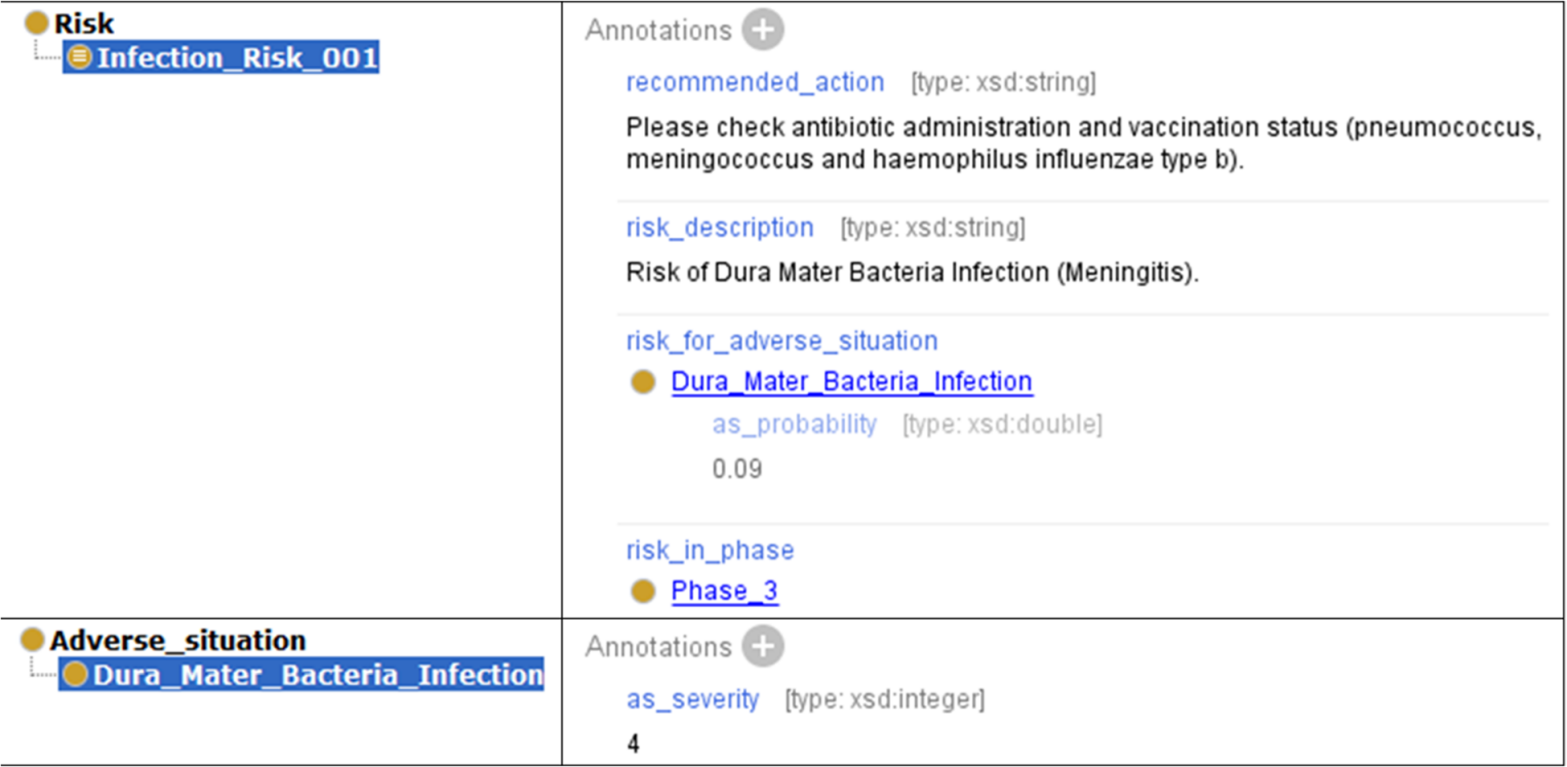
Annotations of risk and adverse situation

The tool RIOGen, developed within the project, generates ontological entities from the risk specification and inserts them into RIO. For every risk condition, for example, a subclass of the corresponding KPI is inserted. Here the class names are automatically generated according to certain rules. For every condition class an anonymous equivalent class is created as property restriction, based on the property has_data_value (Fig. 5). Then, for each risk a subclass of rio:Risk is created. The name for the subclass is defined in the risk specification (e.g., Risk Name: Infection_Risk_001, Fig. 4). For the risk subclass, an equivalent anonymous class is also defined which is based on the has_part property and on the corresponding condition classes; this anonymous class represents the risk specification rule (Fig. 6). Furthermore, the treatment phases are created and connected with those KPIs and risks which are relevant for them. Finally, we define the connections between risks and those adverse situations, which possibly evolve from them, as annotations (incl. probability and severity, Fig. 7). We specified the probability as annotation (as_probability) of the annotation relating to the adverse situation (risk_for_adverse_situation).

### Ontology-based Risk Detector (OntoRiDe)

We developed an ontology-based software module, called Ontology-based Risk Detector (OntoRiDe), which allows the identification of the ontologically specified risks. This tool receives the KPIs of the current potential risk situation as an input parameter, and carries out the risk specification rule, which is contained in the ontology; then it classifies the current situation as a risk or non-risk situation and returns the results. If the current KPIs satisfy one of the rules (i.e., at least one risk is recognized) then the considered situation is a risk situation, otherwise it is a non-risk situation.

Further information, which the tool returns to the user, includes the description of the existing risks, the treatment phases, in which the risks are relevant, but also the adverse situations, which may evolve from them (with the probability of occurrence and degree of severity). The most important functionality is the possibility to recognize the risks, but, furthermore, to determine and provide for every recognized risk all combinations of current KPIs that are responsible for every recognized risk. Using this information the user is able to eliminate all of the risks‘causes.

In the following, we briefly sketch the functionalities of the OntoRiDe. For every risk class the corresponding risk specification rule, which is specified as an anonymous equivalent class (Fig. 6), is interpreted and transformed into a disjunctive normal form (by stepwise execution of the de Morgan rules and of the law of distributivity). Any of the conjunctions presents a possible explanation for the risk (e.g., “c1 AND c4 AND c6” and “c3 AND c5 AND c6”, Fig. 4). Then, the single conditions (Fig. 5) are checked, i.e., it is determined whether the current KPI value is included in the specified value range. If all conditions of the conjunction are satisfied, then the corresponding KPIs and further information are provided for the user as explanation.

We did not use a standard DL reasoner. Instead, we implemented suitable functions in OntoRiDe, which are relevant for the specific risk identification problem. Firstly, we want to apply rules, which cannot be easily interpreted by standard reasoners, especially rules which contain mathematical expressions or predefined constants. Such special types of rules are implemented by the OntoRiDe. Secondly, standard reasoners carry out various tasks, such as checking the consistency, classification, and realization. However, most of these standard tasks are not relevant for the identification of risks. This leads to a reduced efficiency of the overall system, if a standard reasoner is utilized for the interpretation of risk specification rules. Finally, OntoRiDe must provide the user with all possible explanations about the existence of a risk in the current situation in an understandable way. The problem of detection and exploration of all possible explanations or justifications of an entailment is a well-known task, for the solution of which there exists several methods and tools [24–26]. Furthermore, there are various investigations about the cognitive complexity and the understanding of the considered justifications [27, 28]. In this context a justification of an entailment is understood to be “*the minimal set of axioms sufficient to produce an entailment*” [24]. In [27, 28] the understandability of justifications and the corresponding reading strategies of OWL users are analyzed. The details of several user studies show that ontology developers find certain justifications very difficult to understand and to work with. We developed a very simple form of explanation, which is understandable for the medical personnel. The OntoRiDe translates the risk specification rules into a disjunctive normal form and checks all conditions of the respective conjunctions. By this procedure all KPI combinations, verified by the rule as true, and the corresponding conditions (value ranges), can be provided for the user in form of understandable explanations (e.g., age < 5 month and vaccination = “no” and antibiotic prevention = false).

In this way, we identify all and only relevant risks in the current situation, as well as provide all possible explanations for them, so that all requirements have been fulfilled. Although the OntoRiDe is not a reasoner, it is sound and complete with regard to our problem.

### Agent system

OntoRiDe is embedded into an agent system, which is developed within the project OntoMedRisk. The purpose of this system is to conveniently access data, that is distributed across various data sources within a hospital in a unified manner. In this way, the agent system derives elementary information for identifying risk situations. The data has to be collected by the agent system and is determined by a set of KPIs. They represent risk-relevant parameters, which have to be monitored by the agent system throughout the entire perioperative treatment process. The collected KPI-related data is provided for the risk identification and analysis in a centralized fashion. The results of those analyses are then forwarded to the medical staff as context-sensitive hints and alerts. The goal of OntoMedRisk is to reduce the risks of adverse situations and complications through early and adequate interventions.

**Fig. 8:**
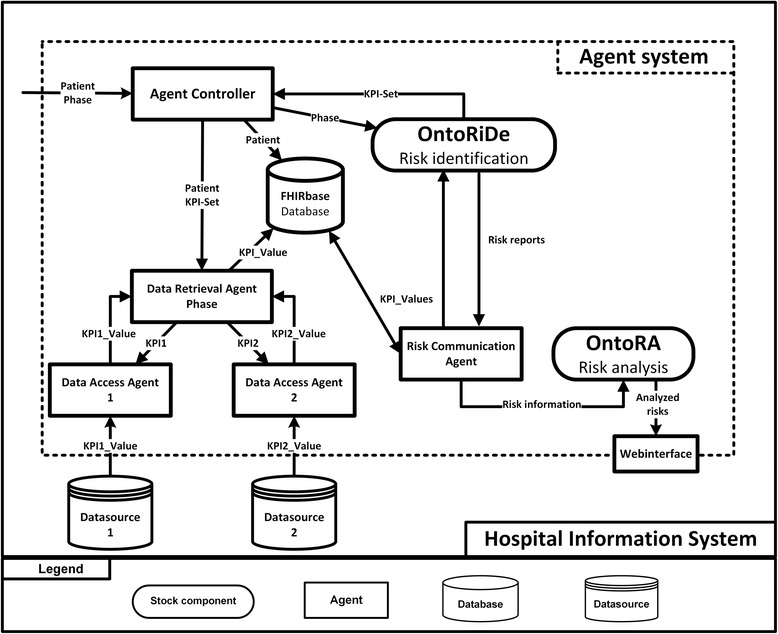
Architecture of the agent system

The functional architecture of the agent system is shown in Fig. 8. The agent system is integrated into the hospital information system from which it collects patient and risk related data. Besides the data and agent related components, the agent system also includes the functional components OntoRiDe and OntoRA (Ontology-based Risk Analysis). The software-based agent system has been implemented using the Java Agent Development Framework (JADE) [29]. JADE embodies a framework, a platform and the middleware for a FIPA-standardized (Foundation for Intelligent Physical Agents, [30]) development of multiagent systems. The main functions of a JADE-based agent system can be categorized into supplying agent behavior and agent communication. The agents communicate in an asynchronous, message-based fashion, using the Agent Communication Language (ACL) [30]. The internal data storage (FHIRbase) of the agent system is based upon the HL7-FHIR specification [31]. Therefore, the data within the agent system is represented as FHIR resources. The agent system models, for example, the information received from OntoRiDe as FHIR RiskAssessment Resource and saves it in the FHIRbase for further analysis. We have been able to map all relevant risk information to FHIR. The input KPIs have been saved, for example, as RiskAssessment.basis (indicates the source data considered as part of the assessment (FamilyHistory, Observations, Procedures, Conditions, etc.)), the possible adverse situations – as RiskAssessment.prediction.outcome (one of the potential outcomes for the patient (e.g. remission, death, a particular condition)), the probability of an adverse situation – as RiskAssessment.prediction.probability (how likely is the outcome), and the explanations for a detected risk – as RiskAssessment.prediction.rationale (additional information explaining the basis for the prediction) [31].

The continuous patient-specific risk monitoring relates to the treatment phases of the perioperative treatment process. Based on the supplied phase information, OntoRiDe provides a phase-specific KPI set to the Agent Controller. Using this information, the Agent Controller generates patient-specific Data Retrieval Agents, which manage the KPI sets and periodically send requests to the Data Access Agents. Those agents are specifically tailored for each data source in order to fetch data correctly. The collected KPI data is sent back to the requesting Data Retrieval Agents and stored in the FHIRbase. Based on a trigger, the Risk Communication Agent fetches the patient-specific KPI data from this database and sends it to OntoRiDe for risk identification purposes. The risk reports resulting from this identification process are then forwarded to OntoRA for further processing. The purpose of OntoRA is to analyze the identified risk situations and to provide the results in a web interface, which can be accessed by medical staff within the hospital information system.

Therefore, OntoRA implements a responsive, web-based user interface hosted on the Apache Tomcat platform [32], which allows the development of a platform-independent solution, lowering costs and increasing flexibility.

**Fig. 9:**
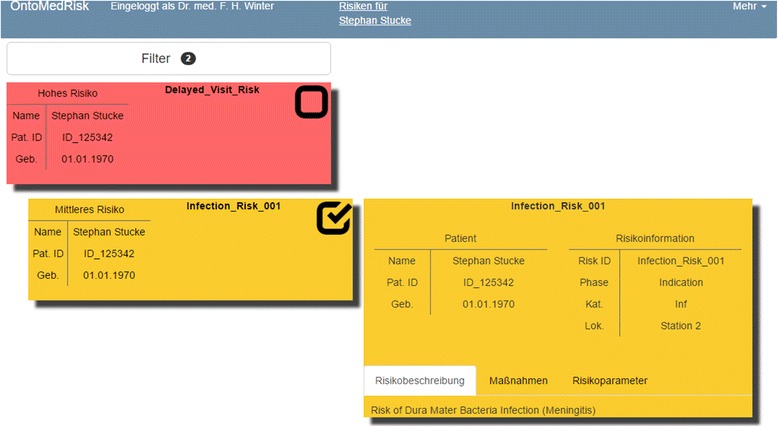
Visualization of risk information in the web interface of OntoRA

The server-sided component of the application consists of two parts, a backend for the web content and a web service to which the agent system can send data. The web service stores the received data in a MongoDB database [33] hosted within the hospital information system. If a client requests data, the backend takes care of this request by fetching the data from the database and sending it to the client. The client-side uses a responsive approach, which allows the usage of web interfaces on multiple devices, such as desktop PCs, tablets, and phones. To achieve this, a combination of HTML5 [34], JQuery [35] and Bootstrap 3 [36] is used. The user interface consists of two web pages, a patient overview and a page containing a patient’s risks, which are displayed in the user’s web browser. The user can select the patient of interest, whose risks are to be displayed. In this view, the risks are ordered by the severity of each risk-event combination. After selecting a risk tile, detailed information like the risk description or risk parameters are displayed (Fig. 9).

**Fig. 10:**
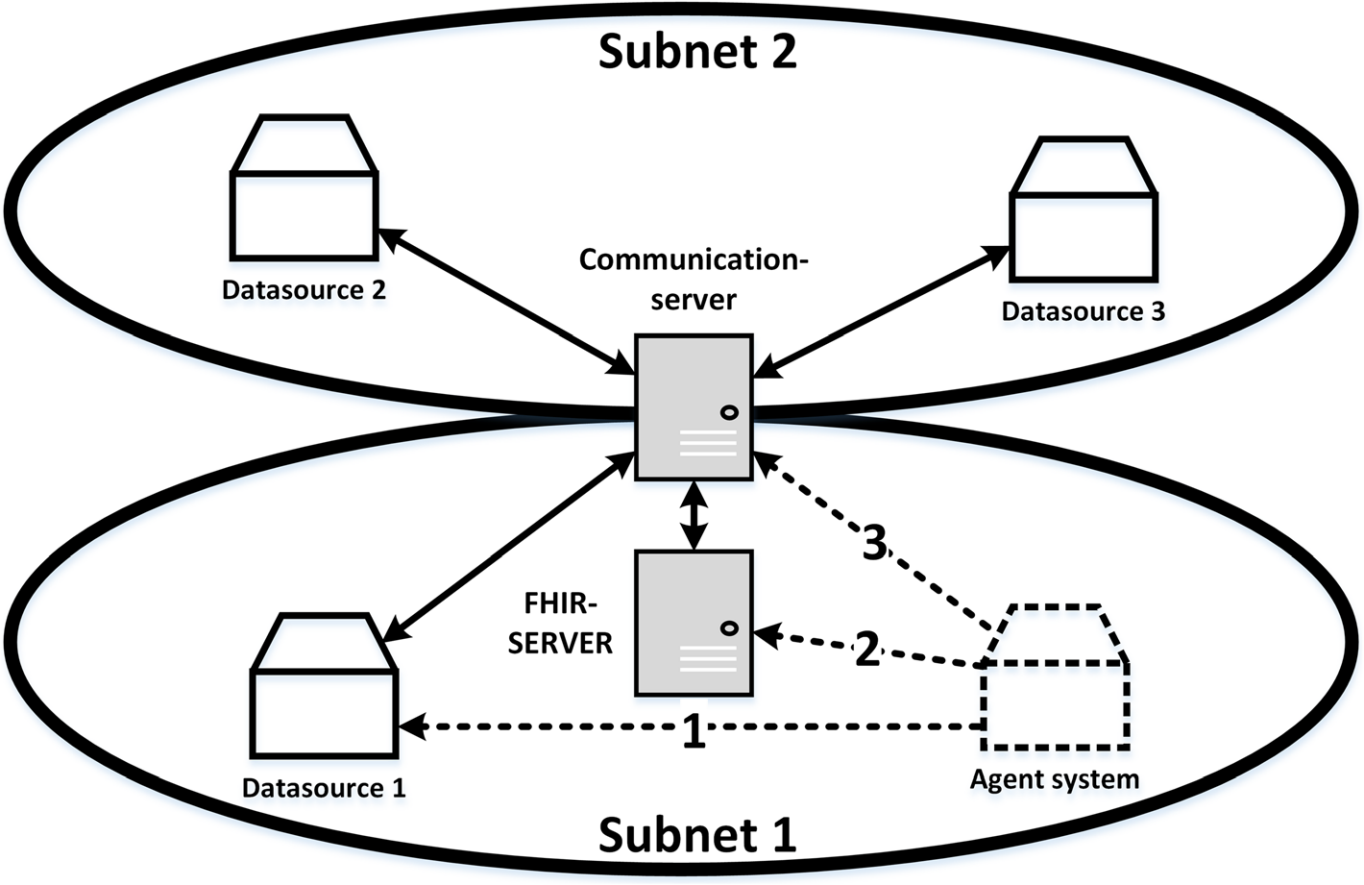
Integration of the agent system into the hospital information system of the Jena University Hospital

The agent system is currently deployed at the Jena University Hospital. Referring to Fig. 8, the hospital information system in which the agent system is integrated into is displayed in Fig. 10. The agent system has to collect data from various data sources within the same subnet (1) and from a FHIR server, which holds patient-related data (2). Because of several linked subnets, the agent system also has to request KPI data from a communication server (3) in order to access data from remote data sources in different subnets.

## Related work

Several approaches towards the formal representation of risks and adverse events through ontologies are described in the literature. We analyzed these existing ontologies for their potential to detect perioperative risks in hospitals, but we concluded that none of these ontologies and tools could be applied to our project.

Bouamrane et al. [37–39] report on the development of an ontology-based system to support clinical decision making. The support is provided in a two-step process. First, the developed system calculates risk scores using numerical formulas. In this step, the system does not use the developed ontology but computes numeric values using an open-source Java-based rule engine (JBoss Rules). After calculating the relevant risk scores, the DL reasoner (Pellet) classifies the patient into several predefined categories for risks, recommended tests and precaution protocols, using the OWL-DL representation of the patient medical history profile and the decision support ontology. The decision support ontology is divided into three domains: a risk assessment ontology, a recommended test ontology and a precaution protocol ontology. The aim of the risk assessment ontology is to detect potential risks of intra-operative and post-operative complications in a given formal representation of a patient medical profile.

Similar to the system of Bouamrane, our approach also provides two components of decision support namely OntoRiDe and OntoRA (Fig. 8). They can perform similar tasks as those of Bouamrane’s system. In addition, OntoRiDe will also use the self-developed RIO for risk identification similarly to the usage of the risk assessment ontology. However, there are also important differences between the two ontologies and systems. The risk assessment ontology focuses only on the patients’ risk related to intra-operative and post-operative complications such as cardio-vascular and respiratory risks, whereas RIO covers various risk types such as special and general treatment risks, technical risks, organizational risks etc. The second significant difference is that our approach integrates the treatment process, its steps, and situations in the risk conceptualization. In this way, it is possible to analyze and identify cross process risks or risk situations so that errors, especially in the perioperative field, could be avoided.

In [40] Third et al. describe a model for representing scientific knowledge of risk factors in medicine. This model enables the clinical experts to encode the risk associations between biological, demographic, lifestyle and environmental elements and clinical outcomes in accordance with evidence from the clinical literature. The major advantage of our approach in comparison with the model developed by Third is the formal representation of cross process risks that can lead to potential adverse situations during different treatment phases. Another added value of our approach is that it can also cover risks related to human and environmental factors such as technical or organizational risks. These types of risks are not considered in Third’s model.

In [41] an ontology of the Open Process Task Model (OPT-Model) is presented. This ontology is primary intended as a generic knowledge base, which implements the various influences of processes and their relations in medical environments, for a prospective risk analysis. The advantage of RIO over the OPT-model-ontology is that it provides an accurate risk analysis. By using RIO, OntoRiDe is able to perform risk classifications according to the risk occurrence time. This process allows us to identify the point in time and treatment phase on which a risk arise. Another further benefit of RIO is the implicitly embedded risk specification, which meets the spirit of evidence-based medicine. This implicit domain knowledge is encoded in OWL rules and can be inferred automatically using ontological reasoning to assess current perioperative risk situations.

In [42] the authors report a clinical decision support system (CDSS) for undergoing surgery based on domain ontology and rules reasoning in the setting of hospitalized diabetic patients. Similar to our approach this system uses logical rules to complement the domain knowledge with implicitly embedded risk specification and clinical domain knowledge. The important upside of our approach is that it does not make restrictions based on certain diseases such as diabetes mellitus, whereas CDSS focuses only on glycemic management of diabetic patients undergoing surgery.

The Ontology of Adverse Events (OAE) [43] and the Ontology of Vaccine Adverse Events (OVAE) [44] (Marcos, Zhao, and He 2013), which was developed based on OAE, describe data relating to adverse events. The OAE was designed to standardize and integrate data relating to adverse events that occur after medical intervention. The OVAE is used for representing and analyzing adverse events associated with US-licensed human vaccines. In OAE the notion adverse event is defined as a pathological bodily process that occurs after a medical intervention (e.g., following a vaccination), while a risk is represented by a factor associated with the occurrence of an adverse event. The work presented here focuses instead on the risk situations and proposes a generic model for the risk specification in the perioperative area. Thus, we do not restrict ourselves to risks that are causally and exclusively related to medical interventions. Contrary to OAE, our approach also considers other risk types such as technical and organizational risks. Moreover, we use the term “adverse situation” in order to avoid excluding situations that are not related to medical interventions.

We also analyzed several conversion tools such as Excel2OWL, Mapping Master and Populus [45–47] for their potential to build an expressive formal ontology from our risk specification spreadsheet, but we concluded that none of these tools could be applied to our project. In fact, our Excel spreadsheet contains domain specific logical rules (see Figs. 4 and 6) that are not covered in these software solutions. We therefore decided to develop RIOGen, a Java tool that enables us to automatically generate RIO entities from the risk specification template.

## Discussion

We elaborated an ontological foundation of the notion of risk, upon which we developed a Risk Identification Ontology (RIO). With help of RIO perioperative risks can be specified, whereas OntoRiDe can be used to identify risks in a given treatment situation. This allows the recognition of risk situations and supports the avoidance of possible adverse effects or consequences. Furthermore, we implemented an agent system to realize the ontology-based approach. This agent system gathers during the whole perioperative treatment process risk-relevant data from various sources and provides it for the risk identification respectively the risk analysis in a centralized fashion. The results of those analyses are transmitted to the medical personnel in form of context sensitive hints and alerts.

None of the presented approaches (s. “Related work”) can answer competency questions such as “Which treatment situation could be a potential risk situation?”, “Which properties or KPIs are responsible for an actual risk situation?” and “Which risk situation belongs to which treatment phase?”. The aim of RIO and OntoRiDe is to solve this issue.

Our approach has the following limitations: 1. Only known und specified risks can be identified by the system; 2. All required data (KPIs) must be available in the respective source systems in electronic form. Therefore, the system can only react to known and correctly specified risks to which the required data was electronically recorded.

## Future work

Further development of the agent system will encompass the implementation of interfaces for different 3rd party data sources in collaboration with their original vendors. To facilitate the expansion of the agent system, a developer package for Data Access Agents will be released, providing interfaces for integrating additional data sources in conformance to the given specifications. Furthermore, it is intended to expand and to optimize the application of the agent system to cater for additional use cases and to better support mobile devices in order to provide real-time feedback and improve usability. Finally, future work could include a machine-learning approach, where the agent system recognizes adverse events by itself and derives risks, which subsequently will be monitored to prevent the repeated occurrence of these adverse events.

The presented Risk Identification Ontology could be used for the ontology-based analysis of clinical studies for different medical applications and use cases. Future work will include further analysis and clinical evaluation studies.

Our present work raises the question of what are the formal, ontological connections between a risk, its adverse situation and its probability. This question will also be examined and discussed in the future.

## Conclusion

We developed the Risk Identification Ontology and an ontology-based agent system, which can identify and analyze risks across medical processes and supports the avoidance of errors in the perioperative setting. About 20 risks relating to cochlear implantations have already been implemented. Comprehensive testing has shown that a stable and platform-independent deployment of all components on different virtual machines was successful. Further testing using the FHIR server as a source for KPI data has illustrated the correctness of the data collection, risk identification and risk analysis components, as well as the web-based visual representation of results. The test system contains a web-based form for entering the test data sets, which are then stored on the FHIR server. The domain experts (medical staff) have tested the functionality and usability of the system based on practice-relevant test data. According to the interviews with domain experts, the system currently meets all specified requirements.

## Abbreviations

ACL: Agent Communication Language
CDSS: Clinical decision support system
CI: Cochlear implantation
CIRS: Critical Incident Reporting System
EPC: Event-driven process chain
FHIR: Fast Healthcare Interoperability Resources
FIPA: Foundation for Intelligent Physical Agents
GFO: General Formal Ontology
IOM: Institute of Medicine of the US National Academy of Sciences
JADE: Java Agent Development Framework
KPI: Key Performance Indicator
OAE: Ontology of Adverse Events
OntoRA: Ontology-based Risk Analysis
OntoRiDe: Ontology-based Risk Detector
OPT-Model: Open Process Task Model
OVAE: Ontology of Vaccine Adverse Events
PRS: Potential risk situation
PTRD: Point in time of risk detection
RIO: Risk Identification Ontology
